# Anti-Inflammatory Activity of *N*′-(3-(1H-indol-3-yl)benzylidene)-2-cyanoacetohydrazide Derivative via sGC-NO/Cytokine Pathway

**DOI:** 10.3390/ph16101415

**Published:** 2023-10-05

**Authors:** Pablo Rayff da Silva, Nadjaele de Melo Apolinário, Simone Ângela Soares da Silva, Maria Elaine Cristina Araruna, Thássia Borges Costa, Yvnni M. S. de Medeiros e Silva, Teresinha Gonçalves da Silva, Ricardo Olímpio de Moura, Vanda Lucia dos Santos

**Affiliations:** 1 Programa de Pós Graduação em Ciências Farmacêuticas, Universidade Estadual da Paraíba, Campina Grande 58429-500, PB, Brazil; pablo-rayff@hotmail.com (P.R.d.S.); nadjaelemelo@gmail.com (N.d.M.A.); simoneangelasoares@gmail.com (S.Â.S.d.S.); elaine.araruna@gmail.com (M.E.C.A.); thassiacosta5@gmail.com (T.B.C.); yvnnim@gmail.com (Y.M.S.d.M.e.S.); vandaluciasantos16@gmail.com (V.L.d.S.); 2 Laboratório de Ensaios Farmacológicos, Departamento de Farmácia, Universidade Estadual da Paraíba, Campina Grande 58429-500, PB, Brazil; 3 Laboratório de Desenvolvimento e Síntese de Fármacos, Departamento de Farmácia, Universidade Estadual da Paraíba, Campina Grande 58429-500, PB, Brazil; 4 Departamento de Antibióticos, Centro de Biociências, Universidade Federal de Pernambuco, Recife 50740-520, PE, Brazil; teresinha100@gmail.com

**Keywords:** cytokines, leukocyte migration, *N*-acylhydrazones, nitric oxide, JR19

## Abstract

The *N*-acylhydrazone function has been reported as a pharmacophore group of molecules with diverse pharmacological activities, including anti-inflammatory effects. Therefore, this study was designed to evaluate the anti-inflammatory potential of the compound *N*′-(3-(1H-indol-3-yl)benzylidene)-2-cyanoacetohydrazide (JR19) in vivo. The study started with the carrageenan-induced peritonitis model, followed by an investigation of leukocyte migration using the subcutaneous air pouch test and an assessment of the antinociceptive profile using formalin-induced pain. A preliminary molecular docking study focusing on the crystallographic structures of NFκB, iNOS, and sGC was performed to determine the likely mechanism of action. The computational study revealed satisfactory interaction energies with the selected targets, and the same peritonitis model was used to validate the involvement of the nitric oxide pathway and cytokine expression in the peritoneal exudate of mice pretreated with L-NAME or methylene blue. In the peritonitis assay, JR19 (10 and 20 mg/kg) reduced leukocyte migration by 59% and 52%, respectively, compared to the vehicle group, with the 10 mg/kg dose used in subsequent assays. In the subcutaneous air pouch assay, the reduction in cell migration was 66%, and the response to intraplantar formalin was reduced by 39%, particularly during the inflammatory phase, suggesting that the compound lacks central analgesic activity. In addition, a reversal of the anti-inflammatory effect was observed in mice pretreated with L-NAME or methylene blue, indicating the involvement of iNOS and sGC in the anti-inflammatory response of JR19. The compound effectively and significantly decreased the levels of IL-6, TNF-α, IL-17, and IFN-γ, and this effect was reversed in animals pretreated with L-NAME, supporting a NO-dependent anti-inflammatory effect. In contrast, pretreatment with methylene blue only reversed the reduction in TNF-α levels. Therefore, these results demonstrate the pharmacological potential of the novel *N*-acylhydrazone derivative, which acts through the nitric oxide pathway and cytokine signaling, making it a strong candidate as an anti-inflammatory and immunomodulatory agent.

## 1. Introduction

The inflammatory process is defined as a natural response of the immune system to noxious stimuli affecting cells or tissues triggered by chemical, physical, or biological agents [1,2]. When activated excessively and persistently, this process can lead to organ and system damage associated with the pathogenesis of various diseases, including autoimmune diseases, cancer, and musculoskeletal, gastrointestinal, and psychoemotional disorders, as well as worsen the prognosis of viral infections, thus contributing to healthcare costs [3,4,5].

The pathophysiology of inflammation involves vasodilation resulting from the release of mediators by leukocytes that migrate from the blood to the site of injury, leading to characteristic symptoms such as edema, pain, and redness. If allowed to continue without intervention, this process may result in the deterioration of organ or tissue function [6]. Effector mechanisms of the immune system include the expression of cytokines, eicosanoids, vasoactive amines, and nitric oxide, which play a role in signaling and activating vascular endothelial cells and higher regulatory adhesion molecules that mediate leukocyte rolling, cellular adhesion, and extravasation to the site of inflammation, actively contributing to the cellular response [2].

An essential mediator in the onset of inflammation is nitric oxide (NO), a small signaling molecule whose actions are primarily controlled by the expression and activities of nitric oxide synthase (NOS) enzymes: neuronal (nNOS), endothelial (eNOS), or inducible (iNOS). These enzymes facilitate NO synthesis by converting L-arginine to L-citrulline [7,8]. The exact role of NO remains unknown due to its dual properties as both an anti-inflammatory and pro-inflammatory agent. However, the expression of iNOS is known to be induced by inflammatory responses [8].

In localized infections and sepsis, iNOS expression is associated with inhibition of pathogen growth and platelet aggregation, and the NO pathway is involved in peripheral analgesia in primary sensory neurons [7,8]. In addition, iNOS can be transcriptionally stimulated by activation of TLRs (Toll-like receptors) in response to PAMPs (pathogen-associated molecular patterns), where biosynthesized NO exerts its vasoactive function in acute inflammatory responses accompanied by leukocyte migration throughout the process [9].

Another critical event in the pathophysiology of inflammation is the biosynthesis and release of prostaglandins (PGs). The pharmacological action of classical nonsteroidal anti-inflammatory drugs (NSAIDs) is to suppress the biosynthesis of these mediators from arachidonic acid by selective or non-selective inhibition of cyclooxygenase (COX) enzymes [2,10]. In contrast to steroidal anti-inflammatory drugs (SAIDs), the extended usage of non-steroidal anti-inflammatory drugs (NSAIDs) is generally considered safer. NSAIDs are on the World Health Organization Model List of Essential Medicines [11].

Non-selective NSAIDs in conventional therapy, although effective, have undesirable adverse effects on the gastrointestinal (GI) tract, renal and hepatic systems due to the constitutive expression of cyclooxygenase 1 (COX-1) in the body, which serves as a source of cytoprotective PGs for the GI tract [8,11]. Therefore, the development of drugs with selective inhibition of cyclooxygenase 2 (COX-2) has been proposed, assuming greater therapeutic advantages since it is an isoform induced in response to the onset of an inflammatory process and associated with various pathological dysfunctions [12]. However, these drugs may have serious cardiovascular side effects due to the suppression of cardioprotective PGs derived from COX-2 [13].

Thus, the critical role of iNOS and COX-2 enzymes and cytokines in the initiation and progression of inflammation is evident. Therefore, drugs capable of modulating these targets and associated with a low toxicity profile are essential as potential pharmacological agents for new anti-inflammatory therapies [14,15]. In this context, acylhydrazone derivatives stand out as a class of synthetic chemical structures with diverse bioactivity, among which the *N*-acylhydrazone function has shown relevant anti-inflammatory and analgesic responses [14]. Therefore, this study aims to evaluate the in vivo anti-inflammatory potential of the compound *N*′((1*H*-indol-3-yl)methylene)-2-cyanoacetohydrazide (JR19) and to perform in silico and in vivo investigations of possible mechanisms of action involved in the anti-inflammatory response in terms of cellular behavior and inflammatory cytokine expression.

## 2. Results

### 2.1. Carrageenan-Induced Peritonitis

**Figure 1:**
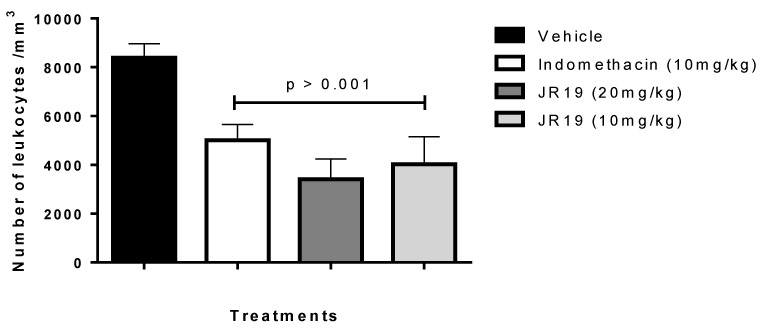
Influence of JR19 (10 and 20 mg/kg) treatment on total leukocyte count in the peritonitis induced by carrageenan 1%. Values are expressed as mean ± standard deviation of the mean, *n* = 6. *p* > 0.001 significantly different from vehicle group (saline) (ANOVA followed by Dunnett’s test).

This study aimed to evaluate the anti-inflammatory potential of the *N*-acylhydrazone derivative JR19. For this purpose, the carrageenan-induced peritonitis test was performed. Carrageenan is a polysaccharide of plant origin isolated from red algae and widely used as an inflammatory agent; thus, it chemically activates the inflammatory process by triggering different pathways of the inflammatory cascade [16]. In this experimental model, oral administration of JR19 (at doses of 10 and 20 mg/kg) significantly inhibited leukocyte migration into the peritoneal cavity by 59% and 52%, respectively. Standard treatment with indomethacin (10 mg/kg) resulted in 40% inhibition. All groups were compared to the vehicle (saline) group. Since there was no significant difference between the 10 mg and 20 mg doses of JR19, the 10 mg/kg dose was selected for further pharmacological testing (Figure 1).

### 2.2. Subcutaneous Air Pouch

**Figure 2:**
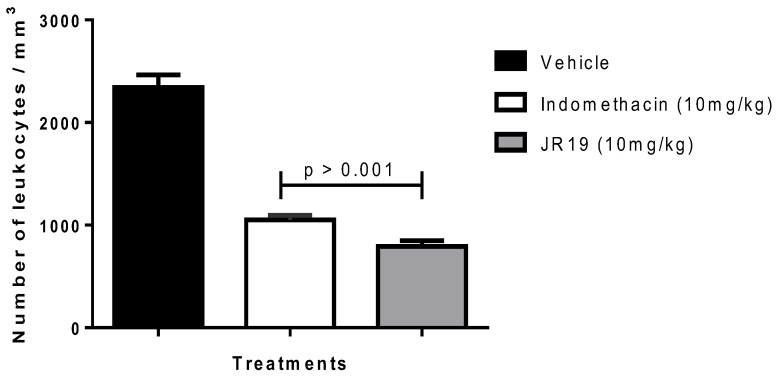
Influence of JR19 (10 mg/kg) treatment on total leukocyte count in the subcutaneous air pouch induced by carrageenan 1%. Values are expressed as mean ± standard deviation of the mean, *n* = 6. *p* > 0.001 significantly different from vehicle group (saline) (ANOVA followed by Dunnett’s test).

The subcutaneous air pouch experimental model is of great value in the assessment of acute inflammation. Administration of carrageenan into the air pouch induces an immediate inflammatory response, resulting in elevated levels of prostaglandins (PGs) and leukotrienes (LTs) in the exudate [17]. This model effectively mimics rheumatoid arthritis, making it an invaluable screening method for potential arthritis treatment candidates. The air pouch formed on the back of the mice closely mimics the inflamed synovium observed in patients with the disease [18,19]. In this model, JR19 demonstrated anti-inflammatory activity, as indicated by a significant 66% reduction in cell migration compared to the vehicle group. The standard drug (indomethacin) used in this model was tested at its therapeutic dose of 10 mg/kg and showed 55% inhibition (Figure 2).

### 2.3. Formalin-Induced Nociception

**Figure 3:**
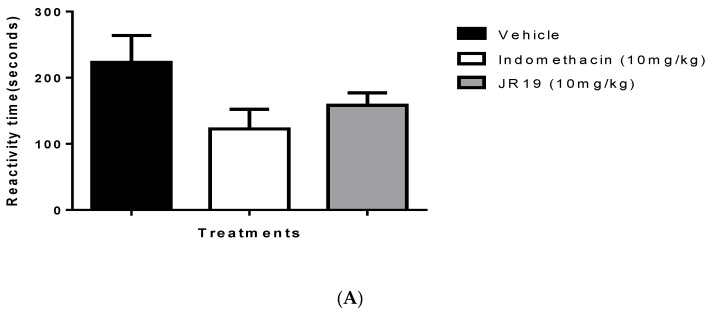

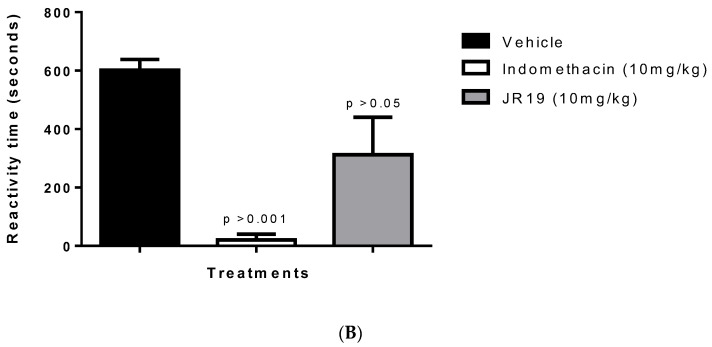
Influence of treatment with the compound JR19 on the time of reactivity to pain in phase I (**A**) and phase II (**B**) of the intraplantar formalin test. Values are expressed as mean ± standard deviation of the mean, *n* = 6. *p* > 0.05 or *p* > 0.001 significantly different from vehicle group (saline) (ANOVA followed by Dunnett’s test).

The formalin-induced nociception model was used as a starting point for the study of antinociceptive effects. This model allows the identification of drug candidates with analgesic effects dependent on anti-inflammatory action and involves two phases of nociceptive behavior that indicate the involvement of different chemical mediators. The first phase is characterized by profound neurogenic pain and involves chemical mediators such as P, glutamate, and bradykinin. The subsequent phase appears to result from changes in tissue and function within the dorsal horn of the spinal cord and involves agents such as histamine, serotonin, prostaglandins, and bradykinin. Crucially, the second phase does not occur due to the first one. There is a period of quiescence between the two phases in which no stimulus is manifested [20,21]. Oral administration of JR19 or indomethacin at a dose of 10 mg/kg did not affect this model’s first phase of nociception (neurogenic pain) (Figure 3A). In the second phase of nociception (inflammatory pain), both JR19 and the standard drug group, indomethacin, significantly reduced the animals’ response time to intraplantar formalin injection by 39% and 93%, respectively, compared to the vehicle group (Figure 3B).

### 2.4. Molecular Docking

Because of the results of in vivo studies presented previously, the possible involvement of JR19′s mechanism of action in the oxide nitrergic pathway was initially investigated through in silico analysis. In this way, the selected targets were inducible nitric oxide synthase co-crystallized with AR-C95791 (PDB ID: 3E7G), IkBb/NFkB p65 homodimer complex (PDB ID: 1K3Z) and soluble guanylate cyclase 1 (PDB ID: 3UVJ). The scores achieved by each ligand analyzed are described in Table 1, where higher scores mean better interactions inside the respective binding site, demonstrated in Table 2.

**Figure 4:**
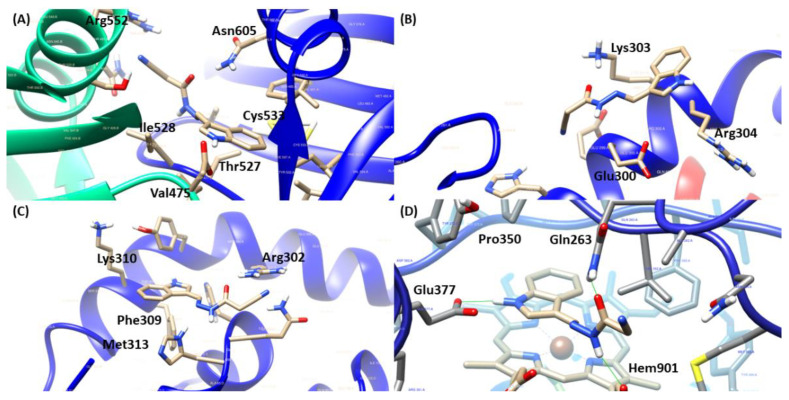
3D representation of JR19 in sGC (**A**), NFκB (**B**)-pose 1, NFκB (**C**)-pose 2 and iNOS (**D**) binding site.

Regarding the target sGC, a nitric oxide sensor that, when activated, performs the catalytic conversion of GTP to cGMP, propagating downstream signals. The reference compound, methylene blue, presented important interactions with Asp530 and Asp486 residues essential for metal coordination surrounding the ATP binding site. However, JR19 demonstrated another approach of inhibition, given the interaction with Arg552 (Figure 4), which is responsible for the stabilization of γ-phosphate from the substrate [22]. Moreover, JR19 exhibited a higher score than methylene blue, suggesting that the additional interactions through hydrogen bonds with Arg552 and Thr527 were involved in that achievement.

For the transcription factor (NFκB), there were two different poses of JR19 inside the binding site (Figure 4), which possessed equivalent scores in comparison to the reference compound nigakinone mainly through hydrogen bonds and transfer charge interactions, where JR19 interacted with Arg302 and Arg304 (also observed for nigakinone) that are two basic residues from the p65 NLS for ion pairing interactions with acidic residues in IκBβ, contributing to maintaining the stability of NFκB-Iκβ complex, therefore preventing the NFκB’s activation [23]. Additionally, in the studies reported by Qu and collaborators [24], constituents from an ethyl acetate extract that significantly suppressed LPS-induced increases in NfκB p65 in the nucleus also interacted with Arg302, Arg304 and Phe309 residues in a similar way as JR19.

Concerning the iNOS target, crucial to the inflammatory process due to the stimulation of immune cells by pro-inflammatory cytokines and endotoxins, its inhibition controls the deleterious effects mediated by NO [25]. Moreover, JR19 presented a higher score value than the inhibitor of iNOS, L-NAME, and was close to the result of the cocrystallized inhibitor (IC_50_ = 0.35 μM). According to Garcin and collaborators [26], the amino acid residues Gln263 and Glu377 are essential for the inhibitory activity of the target, given that substitutions lead to binding affinity reductions (Kd), mostly Glu 377, whose Kd ranged from 0.4 µM to above 100 µM. In this way, besides the interactions with Gln263 and Glu377, JR19 established a parallel position in relation to the heme group (Figure 4), favoring the interaction with the cofactor Hem901, additionally interacted with Pro350 that was also observed for the ligand cocrystallized and L-NAME.

### 2.5. Investigation of the Participation of the Oxidonitrergic Pathway in the Anti-Inflammatory Effect of JR19

**Figure 5:**
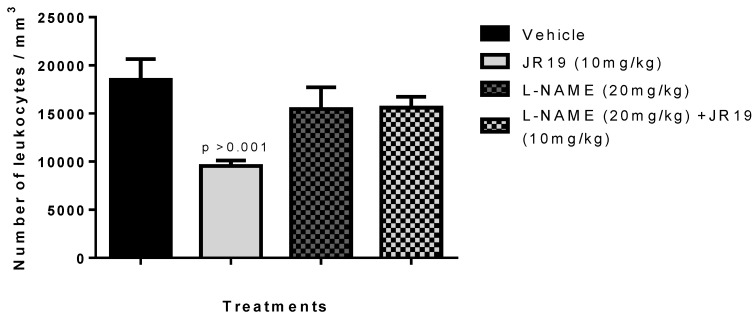
Influence of treatment with the compounds JR19 and L-NAME on the total leukocyte count in the model of peritonitis induced by carrageenan (1%). Values are expressed as mean ± standard deviation of the mean, *n* = 6. *p* > 0.01 or *p* > 0.001 significantly different from vehicle group (saline) (ANOVA followed by Dunnett’s test).

To evaluate the involvement of NO in the anti-inflammatory mechanism of compound JR19, the carrageenan-induced peritonitis experiment was performed using an iNOS inhibitor. The results showed that pretreatment of animals with L-NAME reversed the anti-inflammatory effect of compound JR19 on leukocyte migration (15.6%). Thus, the animals treated with L-NAME and the compound did not show a decrease in the number of leukocytes similar to the group treated with the compound alone (48.3%), indicating that the maintenance of NO synthesis is a crucial factor in the anti-inflammatory activity of JR19 (Figure 5). However, treating animals with L-NAME alone did not show a statistically significant difference compared to the vehicle group in this experiment. These results suggest that NO produced by the activation of iNOS is associated with the suppression of leukocyte migration induced by the compound.

**Figure 6:**
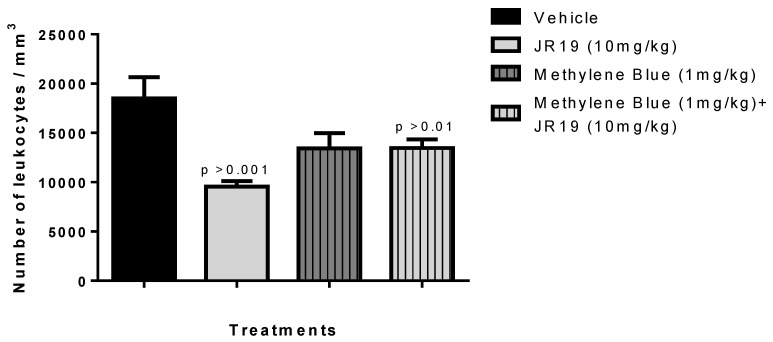
Influence of treatment with the compounds JR19 and Methylene Blue on the total leukocyte count in the model of peritonitis induced by carrageenan (1%). Values are expressed as mean ± standard deviation of the mean, *n* = 6. *p* > 0.01 or *p* > 0.001 significantly different from vehicle group (saline) (ANOVA followed by Dunnett’s test).

Concerning experimental pretreatment with the non-specific soluble guanylate cyclase inhibitor methylene blue (1 mg/kg, i.p.), a significant reversal of the inhibitory effect on leukocyte migration promoted by the compound JR19 (10 mg/kg, p.o.) was observed. Moreover, the administration of the inhibitor alone did not produce any significant modifications in the response to carrageenan compared to the vehicle group (Figure 6). This suggests that when used in isolation, this agent does not elicit changes in the animals’ baseline inflammatory response to carrageenan. These results support the involvement of NO in the activity of compound JR19 and suggest that the mechanism of action of JR19 may involve the maintenance of NO levels in the acute inflammation model tested in this study.

### 2.6. Quantification of Cytokines in Peritoneal Exudate

Cytokines are essential mediators that direct the inflammatory response to sites of infection and injury. At elevated concentrations, cytokines lead to the activation of nuclear transcription factors such as NFκB, which is associated with inflammation and progression of the process [27]. Therefore, we evaluated the effects of compound JR19 on the production of pro-inflammatory cytokines (TNF-α, IL-2, IFN-γ, IL-6, and IL-17), which play a crucial role in leukocyte recruitment, as well as the anti-inflammatory cytokine (IL-4) in the carrageenan-induced peritonitis model in the presence or absence of L-NAME or methylene blue.

**Figure 7:**
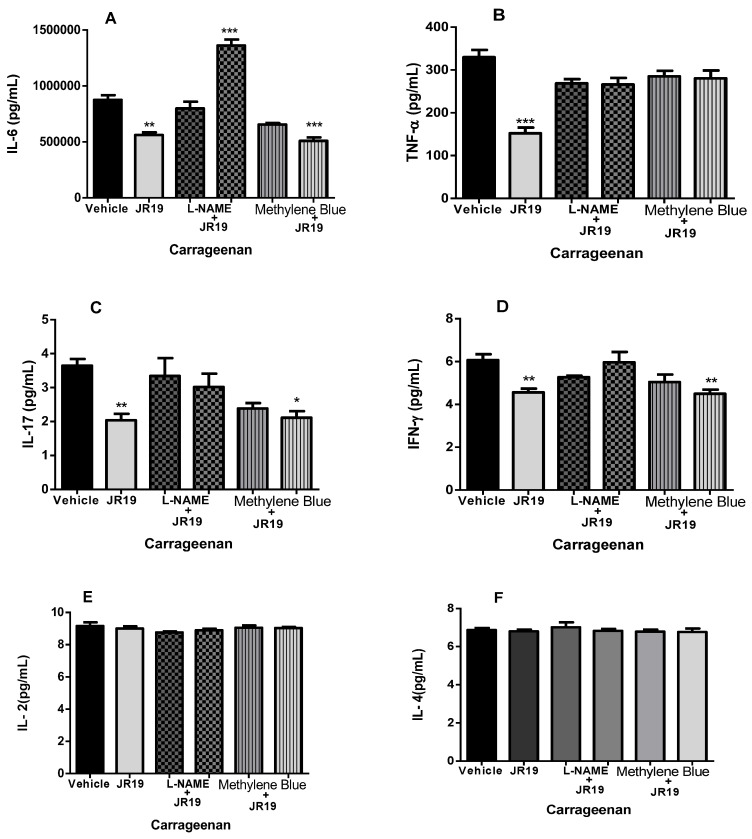
(**A**–**F**). Effect of JR19 compound and pretreatment with L-NAME or methylene blue (i.p.) administered 30 min before JR19 compound (p.o.) on the concentrations of cytokines (IL-6, TNF-α, IL-17, IFN-γ, IL-2, and IL-4) in the peritoneal exudate of mice. Values are expressed as mean ± SD, and asterisks indicate statistically significant differences compared with the vehicle group (saline). *** *p* > 0.001, ** *p* > 0.01, * *p* > 0.05. Analysis of variance (ANOVA) followed by Bonferroni test.

The compound JR19 (at a dose of 10 mg/kg) significantly decreased the levels of the pro-inflammatory cytokines IL-6 (Figure 7A), TNF-α (Figure 7B), IL-17 (Figure 7C), and IFN-γ (Figure 7D). This effect was reversed in groups of animals pretreated with L-NAME, supporting the NO-dependent anti-inflammatory effect of the compound, which, to some extent, contributes to the reduction in leukocyte numbers. In contrast, a reversal of the reduction in TNF-α levels was observed in the groups pretreated with methylene blue (Figure 7B). The compound did not affect the levels of IL-2 (Figure 7E) and IL-4 (Figure 7F). We also observed that L-NAME or methylene blue administered alone did not cause significant changes in the levels of any of the cytokines studied. These data suggest that the activity of JR19 in reducing the levels of these cytokines may depend on maintaining NO levels but may be independent of sGC enzyme activation.

## 3. Discussion

With the rise of medicinal chemistry, new carbon scaffolds have shown potential for various pharmacological activities. Based on this premise, the JR19 molecule was previously designed and synthesized from *N*-acylhydrazone scaffolds associated with indolic groups—an important pharmacophore for anti-inflammatory and immunomodulatory activity as they act on targets such as phospholipase A2, COX-2, and cytokines. This new derivative may provide an alternative to the non-steroidal anti-inflammatory drugs involving nitric oxide signaling, with preclinical potential in acute inflammatory conditions [28,29,30].

Initially, the peritonitis model was used to study cellular behavior in response to the inflammatory stimulus, using carrageenan as the inflammatory agent. This specific polypeptide stimulates the expansion of capillaries within the peritoneal membrane, resulting in increased blood flow and structural changes within the microcirculatory system. These changes facilitate the leakage of plasma proteins from blood vessels into the interstitial space as inflammatory exudate and trigger the movement of white blood cells from the microcirculation to accumulate at the site of initial injury [31,32]. Substances with anti-inflammatory activity can reduce leukocyte migration into the peritoneal cavity by two mechanisms: by inhibiting the synthesis and/or release of chemotactic mediators or by inhibiting the expression of adhesion molecules, since the presence of chemotactic substances is necessary to facilitate their migration to the site of injury and to trigger their effects in an attempt to eliminate the aggressive agent [33,34].

Leukocytes play a critical role in the defense and repair provided by the inflammatory response, although platelets and erythrocytes also participate. By releasing chemical mediators of inflammation, leukocytes migrate across interendothelial junctions (diapedesis) and move toward sites of inflammation [35]. In addition to their chemotactic effect, these mediators can trigger a cascade capable of amplifying and releasing other stimulatory factors. Leukocyte activation produces arachidonic acid (AA) metabolites, degranulation and secretion of lysosomal enzymes, cytokine secretion, and increased adhesion molecule expression and integrin exposure [35,36].

Thus, animal models of acute inflammation that allow the quantitative assessment of leukocyte migration have been widely used, making it possible to quantitatively measure cellular migration, inflammatory mediators, and plasma extravasation after an acute inflammatory process induced by various irritants applied in the cavity [37,38,39]. The results obtained in this screening suggest that JR19 has anti-inflammatory activity, possibly related to its ability to suppress the action and/or release of vasoactive amines, NO, and PGs. These results are consistent with those described by other authors, such as Silva (2022), who tested a ferrocenyl-N-acylhydrazone derivative (100 μmol/kg) and found it active in this model.

The second model used to investigate the cellular migration profile of JR19 was the subcutaneous air pouch model. The main difference between the air pouch and carrageenan-induced peritonitis models is the predominant cell type in the exudate. In the subcutaneous air pouch model, neutrophils are the most abundant cells responsible for chemotaxis. In contrast, carrageenan-induced peritonitis shows many macrophages and mast cells attracted to the site of inflammation by pro-inflammatory cytokines, particularly IL-1β and TNF-α [40,41]. Based on the results obtained in this study, the tested compounds specifically inhibit the production of mediators by neutrophils and macrophages, considering the significant inhibition observed in the air pouch and peritonitis assays, respectively.

Salomé et al. [42] tested a series of naphtyl-*N*-acylhydrazone derivatives and obtained satisfactory results in the subcutaneous air pouch model. At the doses tested (1, 10, and 30 μmol/kg), most compounds showed statistically similar effects to the standard dexamethasone (6.5 μmol/kg). Similarly, Cordeiro [43] tested doses of 30 and 100 μmol/kg of a new *N*-acylhydrazone derivative and its corresponding hydrochloride, using dexamethasone (65 μmol/kg) as a standard.

Considering the anti-inflammatory potential of JR19, it has been suggested that this derivative may also have an antinociceptive effect induced by formalin through a biphasic response. The two phases of the formalin test have different characteristics, making it useful for evaluating analgesic substances and elucidating the mechanism of analgesia. The first phase is characterized by the immediate transmission of impulses to the central nervous system and occurs during the first 5 min after applying the inflammatory agent. It is sensitive to drugs that interact with the opioid system, such as morphine. This pain is caused by the direct action of formalin, especially on the C-afferent sensory fibers and partially on the Aδ afferent fibers [44,45]. The second stage is more prolonged, occurring 15 to 30 min after formalin injection, and is associated with the development of an inflammatory response. This phase can be suppressed by non-steroidal anti-inflammatory drugs (NSAIDs) such as indomethacin. Thus, centrally-acting drugs (opioids) can suppress both phases, whereas peripherally-acting drugs only inhibit the second phase [20,21,46].

Compound JR19 was active only in the second phase of formalin administration, confirming its lack of central analgesic effect and possibly its retention of anti-inflammatory activity. This effect may be attributed to the inhibition of the synthesis of inflammatory mediators such as PGs, TXs, and LTs [41]. Thus, the observed antinociceptive effect involves peripheral mechanisms acting on inflammatory pain. These results support the hypothesis that the *N*-acylhydrazone derivative JR19 may have peripheral antinociceptive activity, possibly by inhibiting PG synthesis or by inhibiting the release and/or production of inflammatory mediators, such as cytokines, histamine, and serotonin, among others. A study by Silva (2015) [47] evaluated the antinociceptive activity of a series of new cyclohexyl-*N*-acylhydrazone derivatives. Most compounds tested showed a relevant antinociceptive profile in the formalin-induced inflammatory response (second phase). The compounds active in the neurogenic phase were evaluated in the hot plate test, where the hypothesis of central antinociceptive activity was ruled out.

As demonstrated by molecular docking, the involvement of the enzymes “soluble guanylate cyclase” and “inducible nitric oxide synthase” was investigated using the carrageenan-induced peritonitis model. This model induces sudden inflammation in mice, causing the body to deploy immune system cells, primarily neutrophils, to the site of injury. A critical step in this response is the attachment of circulating leukocytes to the endothelial cells lining the blood vessels, which facilitates their subsequent movement across these cell barriers to the site of inflammation. Leukocyte migration from the circulation to the injured tissue is a critical event in the development of the inflammatory process [35,41]. As leukocytes migrate to the tissue, they release several chemical mediators that can amplify and prolong the inflammatory process. Therefore, compounds capable of inhibiting the activation of these cells can reduce the inflammatory process [48].

The presence of NO in biological systems is often determined based on physiological effects such as vasodilation, activation of sGC, increased cGMP concentration, citrulline production, or inhibition of platelet aggregation [49]. It can also be assessed using NO synthesis inhibitors such as L-arginine analogs or hemoglobin and by measuring nitrite and nitrate concentrations. These methods have varying degrees of specificity and provide indirect information on NO production [50].

Our results suggest that JR19 may exert anti-inflammatory effects by modulating NO levels. NO plays a crucial role in various aspects of the inflammatory response, but its role in leukocyte migration is controversial in the literature, with evidence supporting both anti-inflammatory and pro-inflammatory effects [51]. Studies suggest that NO exerts anti-inflammatory effects by interfering with leukocyte migration. An important piece of evidence for the anti-inflammatory effects of NO is related to the activation of the second messenger cGMP [52,53].

NO promotes the modulation of P-selectin (adhesion protein) expression vitro induced by IL-1β produced in the endothelium through activation of the sGC enzyme, thereby reducing leukocyte adhesion to the vascular wall and neutrophil aggregation and secretion [52]. Elevated cGMP levels are also associated with a reduction in glycoprotein IIb/IIIa expression, another important leukocyte adhesion molecule [54,55]. To support the experimental findings, we also investigated whether the anti-inflammatory activity represented by the reduction of leukocytes exerted by the compound JR19 would involve the sGC-cGMP pathway through blockade of the GCs enzyme by methylene blue, a nonspecific inhibitor of this enzyme.

Iwata et al. [56] and Florentino et al. [57] demonstrated, in carrageenan-induced pleurisy models, that administration of a NO donor (L-arginine) reduces inflammatory cell migration and edema formation. This model, similar to ours, mimics an acute inflammatory process. The anti-inflammatory effect of NO has been attributed to the blockade of transcription factor binding to nuclear DNA (NFκB), thereby inhibiting the synthesis of chemokine IL-8 (a chemotactic factor secreted by activated monocytes and macrophages that promotes the coordinated and directional migration of immune cells such as neutrophils, basophils, and T lymphocytes), thereby compromising the chemotaxis process [52,58].

There is also an essential relationship between NO and O_2_^−^ levels associated with leukocyte migration, which concerns the enzymatic system responsible for O_2_^−^ synthesis, which possesses a heme prosthetic group to which NO can bind, inhibiting the generation of this reactive oxygen species [52,59]. The reduction in O_2_^−^ availability prevents its dismutation to hydrogen peroxide (H_2_O_2_). This chemical species is responsible for inducing leukocyte adhesion by generating PAF (platelet-activating factor) and increasing the expression of other molecules involved in this process [60,61].

In addition, elevated levels of O_2_^−^ can activate NF**κ**B, leading to increased production of cytokines and chemokines and stimulating mast cells to release pro-adhesive agents such as cytokines [62,63,64]. Therefore, we conclude that the anti-inflammatory effect of compound JR19, as observed by the reduction of peritoneal leukocytes, is partly due to the acute maintenance of NO levels.

Through peritonitis using L-NAME or Methylene Blue blockers, iNOS and sGC blockers, respectively, there was an investigation of the expression of cytokines by JR19 (at a dose of 10 mg/kg) by a mechanism dependent on these enzymes. L-NAME is an analogue of L-arginine, the amino acid by which NO is synthesized. It acts as a non-selective and competitive inhibitor of NOS, with an effect on endothelial and cellular function through the regulation of NO biosynthesis [65,66].

Methylene Blue is involved in anti-inflammation by suppressing the iNOS/NO-NF-κB pathway, regulating the eNOS and sGC enzymes responsible for converting GTP to cGMP, leading to vasoconstriction. As for iNOS, it works as a strong inflammatory mediator in different types of cells. It inhibits Sirt1 activation by NO-mediated S-nitrosylation, which activates NFκB and p53 to facilitate inflammatory cytokine expression and apoptosis, respectively [67].

JR19 (at a dose of 10 mg/kg) was effective in significantly reducing the levels of the pro-inflammatory cytokines IL-6, TNF-α, IL-17, and IFN-γ. IL-6 is a cytokine secreted by various cell types such as macrophages, monocytes, eosinophils, and hepatocytes, and TNF-α is a potent inducer of IL-6 [38]. It serves as a pro-inflammatory cytokine that promotes the development and activation of neutrophils and macrophages and supports the differentiation and maintenance of cytotoxic T lymphocytes and natural killer (NK) cells. IL-6 is emerging as a prominent and early mediator in the initiation and regulation of acute phase protein production and secretion by hepatocytes in response to painful stimuli such as infection [59,64]. It also plays a role in the metabolic control of C-reactive protein (CRP), an acute phase protein rapidly elevated during inflammatory responses [68,69]. Elevation of serum IL-6 and CRP levels may cause downregulation of NO production by inhibiting the enzyme nitric oxide synthase [70,71].

TNF-α is mainly produced by macrophages and acts on endothelial cells to promote vasodilation and stimulates chemokines to facilitate the chemotaxis of leukocytes, mainly neutrophils and monocytes [72]. In addition to stimulating their activation, TNF-α stimulates the production of acute-phase proteins and fibrinogen during the inflammatory process [73]. Even at low concentrations, TNF-α induces the expression of adhesion molecules in endothelial cells and stimulates macrophages and other cells to secrete chemokines [74,75].

IFN-γ is primarily produced by NK cells and T lymphocytes and acts synergistically with TNF-α to stimulate chemokine secretion [76]. In addition, IFN-γ induces the expression of intercellular adhesion molecule-1 (ICAM-1) and vascular cell adhesion molecule-1 (VCAM-1), promoting better leukocyte adhesion to the endothelium and facilitating their diapedesis to the site of inflammation [76,77]. It has been reported that this cytokine is involved in neutrophil infiltration in malignant tumors, which typically contain various types of immune cells in addition to cancer cells [78].

IL-17 is a pro-inflammatory cytokine produced by T cells that can activate and mature neutrophils [79]. It also stimulates the production of IL-6 and IL-8 (responsible for the migratory stimulus of immune cells, mainly neutrophils, and increases the expression of adhesion molecules on endothelial cells) [79,80]. These pro-inflammatory cytokines, among others, stimulate the production of other cytokines and other inflammatory mediators, such as inflammatory prostanoids via COX-2 [81].

Freitas [82] has classified N-acylhydrazone derivatives as inhibitors of p38 MAPK. Additionally, Cordeiro [43] demonstrated that these derivatives inhibit the expression of mediators involved in this pathway, such as IL-1β, TNF-α, and NO (at doses of 10, 30, and 100 μmol/kg). Furthermore, Cordeiro [43] found that at the same doses, there was a significant reduction in pain induced by formalin during the second phase compared to the standard group using morphine.

Since cytokines play a critical role in the inflammatory response and are produced by various cell types at the site of injury and by cells of the immune system during the inflammatory process, compounds that reduce the levels of pro-inflammatory cytokines may help to inflammation. Thus, JR19 shows great promise in controlling inflammation by acting on key inflammatory pathways, making it a potential candidate for a new anti-inflammatory drug.

## 4. Materials and Methods

### 4.1. Substances

**Figure 8:**
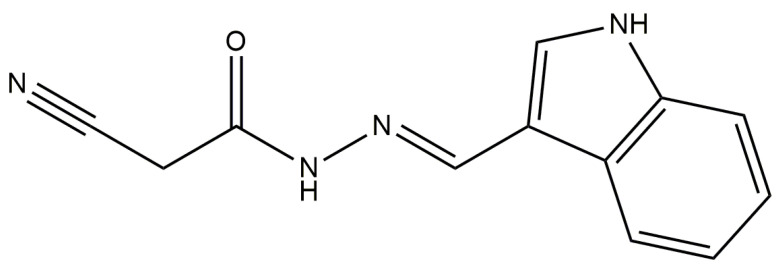
JR19—*N*′((1*H*-indol-3-yl)methylene)-2-cyanoacetohydrazide.

The following substances were used: purified water; aseptic saline (0.9% NaCl); indomethacin (obtained from Sigma Aldrich, St. Louis, MO, USA); carrageenan (obtained from Sigma Aldrich, St. Louis, MO, USA); formalin; sodium heparin (5000 IU/mL, obtained from Cristália^®^, São Paulo, Brazil); ketamine hydrochloride (provided by Vetbrands^®^, São Paulo, Brazil); xylazine hydrochloride (also provided by Vetbrands^®,^, São Paulo, Brazil); Turk’s solution; phosphate-buffered saline (PBS); NG-nitro-L-arginine methyl ester (L-NAME, obtained from Sigma Aldrich, St. Louis, MO, USA); methylene blue; and JR19 (*N*′((1*H*-indol-3-yl)methylene)-2-cyanoacetohydrazide) (Figure 8), which was synthesized and characterized. JR19 was generously provided by the Laboratory of Development and Drugs Synthesis of the State University of Paraíba (UEPB) under Prof. Dr. Ricardo Olímpio de Moura [28].

### 4.2. Biological Activity

#### 4.2.1. Animals

Adult male Swiss (*Mus musculus*) mice weighing 25 to 35 g were used in the study. These mice were obtained from the Keizo Asami Laboratory of Immunopathology (LIKA) of the Federal University of Pernambuco (UFPE). The mice were housed in plastic cages and maintained under controlled environmental conditions, including a temperature of 23 ± 2 °C and a 12-h light-dark cycle. They had ad libitum access to food and water throughout the study, and experiments were performed between 8:00 a.m. and 5:00 p.m. Ethical treatment of the animals was of the highest priority, and strict guidelines were followed. The care and handling of the mice followed the recommendations of the National Institutes of Health Guide for the Care and Use of Laboratory Animals and the Brazilian College of Animal Experimentation guidelines. Before the start of the experiments, the project was approved by the Ethics Committee for the Use of Animals, with registration number 5905022016, at the Center for Higher Education and Development (CESED). Throughout the study, careful measures were taken to ensure the well-being of the animals, with a focus on minimizing suffering, discomfort, and pain until euthanasia.

#### 4.2.2. Inflammatory Models

##### Carrageenan-Induced Peritonitis in Mice

The mice were divided into different groups, including the vehicle group, the standard drug group, and the test groups. In the vehicle group, saline (10 mL/kg) was administered orally. In the standard drug group, indomethacin was administered orally at 10 mg/kg. The experimental groups received two doses of JR19—10 mg/kg and 20 mg/kg. Thirty minutes after treatment, an intraperitoneal injection of 0.25 mL of 1% carrageenan was administered. Four hours after induction of inflammation, the animals were humanely euthanized, and 2 mL of heparinized PBS was introduced into the intraperitoneal cavity to facilitate the collection of exudates. An incision was made, and the exudate was carefully collected. Cells present in the exudate were suspended in 500 μL PBS, and 10 μL Turk’s fluid (diluted 1:20) was added. Leukocyte counts were determined using a Neubauer chamber under light microscopy, focusing on the four outer quadrants [83].

##### Subcutaneous Air Pouch

An injection of sterile air was used to create an air pouch in the back of the mice (group *n* = 6). On the 1st day, 2.5 mL of sterile air was injected, and the procedure was repeated after 72 h. On the 7th day, the mice received vehicle (saline, 10 mL/kg), indomethacin (10 mg/kg), and JR19 (10 mg/kg) orally. To induce inflammation, 1.0 mL of a 1% carrageenan solution was injected into the air pouch one hour after drug administration. Six hours after application of the phlogistic agent, the animals were euthanized, and the pouches were washed with 3.0 mL of PBS at pH 7.2, containing heparin as a carrier liquid. The total leukocyte count was performed in a Neubauer chamber under light microscopy [84,85].

##### Formalin-Induced Nociception

The mice (group *n* = 6) were treated orally with saline (10 mL/kg), indomethacin, and JR19 (10 mg/kg). After 60 min, 20 μL of 2.0% formalin solution was administered under the plantar pad of the right hind paw. The time in seconds that the animals exhibited painful signs with various behaviors was then timed: raising the paw, licking, biting, swinging the injected paw, and reducing the weight on the paw [86]. The reduction in these behaviors is interpreted as an antinociceptive effect. Based on the pattern of responses, it was possible to identify two phases: the first phase (in the first five minutes) and the second phase (fifteen to thirty minutes after injection), which are related to the neurogenic and inflammatory phases, respectively [87,88].

##### Investigation of the sGC/NOS Pathway in the Anti-Inflammatory Effect

The rats were divided into six groups (*n* = 6). Group 1 received saline solution orally; group 2 received compound JR19 (orally, 10 mg/kg); groups 3 and 4 received IP L-NAME (non-specific NOS blocking compounds at a dose of 20 mg/kg) or methylene blue (an sGC blocker at a dose of 1 mg/kg); groups 5 and 6 were first pre-treated with the respective blockers and after 30 min received compound JR19 orally. About 30 min after each treatment, 0.25 mL of 1% carrageenan was injected into the intraperitoneal cavity. Four hours after inducing inflammation, the animals were euthanized, and 2 mL of PBS at pH 7.2 was injected into the peritoneal cavity. The abdomen of the mice was lightly massaged, and through an incision, peritoneal fluids were collected for total leukocyte counting in a Neubauer chamber under light microscopy [83].

##### Dosage of Cytokines in Peritoneal Exudate

The peritoneal exudate was collected and centrifuged at 7000 rpm for 5 min at 4 °C. The supernatant was removed for cytokine assays, namely TNF-α, IFN-γ, IL-2, IL-4, IL-6 and IL-17. For cytokine measurements, we used flow cytometry tubes, a 1 mL multichannel micropipette, 20% tween PBS and a kit for each cytokine for mouse models (BDTM) Cytometric Bead Array (CBA) mouse Th1/Th2/Th17 CBA. The standard protocol provided by the manufacturer for cytokine assays was followed [87].

### 4.3. Molecular Docking

Regarding target selection and preparation, 3D structures of inducible nitric oxide synthase co-crystallized with AR-C95791 (PDB ID: 3E7G), homodimer complex of IkBb/NF-kB p65 (PDB ID: 1K3Z) and soluble guanylate cyclase 1 (PDB ID: 3UVJ) were sourced from the Research Collaboratory for Structural Bioinformatics Protein Data Bank (RCSB PDB—https://www.rcsb.org/) (accessed on 13 March 2022). Co-crystallized ligands, ions, and water molecules were first removed, and hydrogen atoms were added to the structure. Subsequently, these structures were subjected to redocking procedures using the GOLD^®^ v. 5.8.1 software. For redocking, all four algorithms (Chemical Piecewise Linear Potential (ChemPLP), GoldScore, ChemScore, and Astex Statistical Potential—ASP) were applied to obtain FitScores and binding modes. The best binding pose was chosen using a Root-Mean-Square Deviation (RMSD) value ≤ 2Ǻ.

The structures of JR19, nigakinone, N(gamma)-nitro-L-arginine methyl ester (L-NAME), and methylene blue were built using ChemDraw Professional 3D 15.0 software. They were then minimized using ArgusLab^®^ 4.0.1 software, using the AM1 semi-empirical (Austin Model 1), and saved as MOL2 files. Molecular docking investigations were conducted using GOLD^®^ v. 5.8.1 software, using the ChemPLP scoring function. A radius of 6Å around the co-crystallized ligand was selected with the maximum efficiency of the genetic algorithm (GA). As a result, 100 binding poses were generated for each ligand, and the highest FitScore value was further analyzed for interactions with key residues using Discovery Studio^®^ 2021 software. The 3D visualizations were produced using Chimera^®^ 1.17.1 software.

### 4.4. Statistical Analysis

Data are expressed as mean ± standard deviation of the mean (SDM) measurements from six animals within each group. Comparisons between three or more treatments were made using one-way ANOVA followed by Dunnett’s post hoc test or, where appropriate, two-way repeated measures ANOVA followed by Bonferroni’s post hoc test. All data were analyzed using Prism 5.01 software (GraphPad, San Diego, CA, USA). Statistical significance was considered at a threshold of *p* < 0.05.

## 5. Conclusions

Our results showed that JR19 exhibited activity in reducing leukocyte migration and inflammation-related nociception in the inflammatory phase, suggesting that its effects are likely related to the regulation of pro-inflammatory mediators such as nitric oxide and cytokines (IL-6, IL-17, TNF-α, IFN-γ). This hypothesis was supported by in vivo assays and further substantiated by in silico assays where JR19 showed affinity, particularly for iNOS, sGC, and the transcription factor NFκB. Taken together, the data obtained in the screening indicated that the novel *N*-acylhydrazone derivative showed promising anti-inflammatory activity, which opens favorable prospects for further complementary studies to ensure its safety and pharmacological efficacy.

## Figures and Tables

**Table 1:** FitScore values of target-ligand (complexe).

	Molecular Targets			
Compounds	sGC	NFκB/p65	iNOS	RMSD (Å)
**JR19**	55.2887	32.0025 (pose 1) 42.3261 (pose 2)	74.6536	-
**AR-C95791**			82.3597	0.3297
**L-NAME**			66.1212	-
**Nigakinone**		32.5710 (pose 1) 43.4235 (pose 2)		-
**Methylene blue**	46.9947			-

**Table 2:** Interactions inside the binding site of the targets. Types of interactions: (^1^) hydrogen bond; (^2^) charge transfer; (^3^) hydrophobic.

	Molecular Targets			
Compounds	sGC	NFκB/p65 (Pose 1)	NF-κB/p65 (Pose 2)	iNOS
**JR-19**	Arg552 ^1^, Ile528 ^1,3^, Thr527 ^1^, Asn605 ^1^, Val475 ^2,3^ and Cys533 ^2^	Glu300 ^1^, Lys303 ^1,2^ and Arg304 ^3^	Arg302 ^1^, Phe309 ^2^, Met313 ^2^ and Lys310 ^3^	Gln263 ^1^, Hem901 ^1,2^, Glu377 ^1^ and Pro350 ^3^
**AR-C95791**				Glu377 ^1^, Tyr347 ^1^, Asp382 ^1^, Trp372 ^1^, Hem901 ^2,3^, Phe369 ^3^, Val352 ^3^ and Pro350 ^3^
**L-NAME**				Glu377 ^1,2^, Gly371 ^1^, Tyr373 ^1^, Tyr347 ^1^, Trp372 ^1^, Phe369 ^1,3^, Pro350 ^1,3^, Hem901 ^1,2^, Val352 ^3^, Asp382 ^3^
**Nigakinone**		Glu300 ^2^, Lys303 ^1,2^ and Arg304 ^3^	Arg302 ^1^, Tyr306 ^2^ and His68 ^1^	
**Methylene blue**	Asp530 ^1^, Asp486 ^1^, Asn605 ^1^, Ile528 ^1^, Glu526 ^1^, Leu596 ^1^, Ala531 ^3^, Cys533 ^2^, Phe484 ^2^ and Val475 ^3^			

## Data Availability

Data can be requested by contacting the corresponding author.
